# Metabolic and Dietary Factors in Acne Vulgaris and Evaluation of the Acne Vulgaris Treatment with Oral Contraceptive-Based Therapies in Young Adult Women

**DOI:** 10.3390/nu15061488

**Published:** 2023-03-20

**Authors:** Mateusz Kozłowski, Mirela Niedzielska, Anna Lorenz, Agnieszka Brodowska, Ewelina Malanowska, Adam Przepiera, Aneta Cymbaluk-Płoska, Elżbieta Sowińska-Przepiera

**Affiliations:** 1 Department of Reconstructive Surgery and Gynecological Oncology, Pomeranian Medical University in Szczecin, Al. Powstańców Wielkopolskich 72, 70-111 Szczecin, Poland; 2 Department of Endocrinology, Metabolic and Internal Diseases, Pomeranian Medical University in Szczecin, Unii Lubelskiej 1, 71-252 Szczecin, Poland; 3 Department of Gynecology, Endocrinology and Gynecological Oncology, Pomeranian Medical University in Szczecin, Unii Lubelskiej 1, 71-252 Szczecin, Poland; 4 Department of Urology and Urologic Oncology, Pomeranian Medical University in Szczecin, Al. Powstańców Wielkopolskich 72, 70-111 Szczecin, Poland; 5 Pediatric, Adolescent Gynecology Clinic, Department of Gynecology, Endocrinology and Gynecological Oncology, Pomeranian Medical University in Szczecin, Unii Lubelskiej 1, 71-252 Szczecin, Poland

**Keywords:** acne, acne vulgaris, treatment, contraception, sweets, dairy, glucose, insulin, cholesterol, triglycerides

## Abstract

The etiopathogenesis of acne is complex, as several endo- and exogenous factors that affect the sebaceous-hair unit are involved in the development of acne lesions. The main aim of the study was to evaluate selected metabolic parameters before treatment. Another goal of the study was to determine the correlation between selected metabolic and dietary parameters and the severity of acne before treatment. The third objective was to assess the severity of acne before and after treatment, considering the type of treatment used. The final objective was to assess the relationship between the difference in acne severity before and after treatment, considering the type of treatment used and factors of dairy or sweets intake. 168 women participated in the study. The patients belonged to two groups: the study group (99 patients with acne vulgaris) and the control group (69 patients without skin lesions). The study group was divided into subgroups according to the treatment used: contraceptive preparation, contraceptive preparation and cyproterone acetate, and contraceptive preparation and isotretinoin preparation. We found that LDL levels and consumption of sweets correlated with acne severity. The mainstay of acne treatment is contraceptive treatment (ethinylestradiol and drospirenone). The effectiveness of the three contraceptive-based treatments was confirmed by observing the severity of acne. There were no significant correlations between the difference in acne severity before and after treatment with the three treatments and factors of dairy or sweet consumption.

## 1. Introduction

Acne vulgaris (AV) is a chronic inflammatory skin disease. It is considered one of the diseases of civilization due to the significant influence of environmental factors on the severity and incidence of these lesions [1]. The condition affects about 9.4% of the global population. It is usually associated with adolescence but can occur between the ages of 11 and 30. It is believed to affect 80% of people in this age group or even 100% of young people [2,3,4]. The dermatosis usually appears in the second decade of life, becomes less severe with age and resolves at the end of that decade or the beginning of the third. However, there are cases of persistence of the disease until the 30th or even beyond the 40th year of life [5]. Acne lesions are 95% localized on the face and upper torso, but also occasionally in other parts of the body. Due to the localization of the lesions and the chronic nature of the condition, it is often a serious psychological problem for teenage girls and young women [6,7,8]. For this reason, girls and young women with acne are frequent patients of dermatologists, gynecologists, endocrinologists, psychologists, and clients of cosmetology offices.

The etiopathogenesis of acne is complex, as several endo- and exogenous factors that affect the sebaceous-hair unit are involved in the development of acne lesions. The most relevant and documented initiators that influence the formation of acne are genetic conditions, androgen-induced sebum production, colonization of the hair follicle by *Cutibacterium acnes*, and release of mediators during inflammation and keratinization of the hair follicle outlet; dietary habits and lifestyle factors also influence the severity of acne [6,9,10]. It has been shown that environmental factors do not trigger the onset of acne, but rather cause exacerbation of acne lesions. Climatic conditions are documented evidence, as skin lesions in acne patients improve most often during the summer and spring months. Acne vulgaris is more common in urban areas, which may be related to environmental pollution. The potential role of the modern diet in the development of acne lesions has also been studied. So-called insulinotropic foods, especially milk and carbohydrates with a high glycemic index, affect changes in the concentrations of cytokines and insulin-like growth factors; this can activate receptors for androgens and genes that stimulate keratinocyte proliferation and sebocyte lipogenesis. One environmentally, metabolically, and hormonally determined factor is obesity, which, in addition to severe complications in the form of diabetes and cardiovascular disease, causes a higher risk of severe forms of acne in those with a BMI > 27 [11,12]. In turn, low GL (glycemic load) ± low GI (glycemic index) diets reduce free androgens, increase IGFBP-3 (insulin-like growth factor-binding protein 3), and decrease IGF-1 (insulin-like growth factor-1) levels. In this instance, a reduction in the quantity of lesions and the severity of lesions was confirmed [13].

Currently, acne treatment is the domain not only of dermatologists but also of endocrinologists and gynecologists. Hormonal treatment of acne includes anti-androgens (flutamide, spironolactone, anti-androgenic progestogens) and drugs that block ovarian and/or adrenal androgen production (estrogens, cyproterone acetate, GnRH agonists, low-dose glucocorticosteroids, oral contraceptives) [14]. The American Academy of Dermatology (AAD) working group on the treatment of acne vulgaris focuses on topical and systemic therapies (antibiotics and isotretinoin). The authors emphasize limiting the duration of treatment with systemic antibiotics in adults with acne and prescribing concurrent and/or maintenance topical treatments [15]. Isotretinoin, when used orally, reduces sebum secretion, inhibits the production of comedones, reduces skin colonization by *C. acnes*, and exhibits anti-inflammatory effects. Retinoids, derivatives of vitamin A applied to the skin, accelerate the emptying of blackheads and inhibit the formation of new micro-blackheads, weaken the inflammatory reaction, and enhance the penetration of other topical anti-acne preparations. Recommended locally applied retinoids are tretinoin, isotretinoin, and adapalene [16]. Oxybrasion [17] and hydrogen purification [18] have also been used in the treatment of acne vulgaris.

The aim of the study was to evaluate selected metabolic parameters before treatment. Another objective of the study was to determine the correlation between selected metabolic and dietary parameters and the severity of acne before treatment. The third objective was to assess the severity of acne before and after treatment, considering the type of treatment used. The final aim was to evaluate the relationship between the difference in acne severity before and after treatment, considering the type of treatment used and factors of dairy or sweet consumption.

## 2. Materials and Methods

### 2.1. Participation in the Study

#### 2.1.1. Study Subgroups and Treatment Regimens

The study included 168 women aged between 18 and 31 years who were patients of the Gynecological Endocrinology Outpatient Clinic at the Department of Endocrinology, Metabolic Diseases and Internal Medicine at the Pomeranian Medical University in Szczecin and dermatology outpatient clinics. The patients belonged to two groups: the study group (99 patients with acne vulgaris) and the control group (69 patients without skin lesions). The study group was divided into subgroups according to the treatment used:

− OC subgroup—oral contraceptive,
− OC+A subgroup—oral contraceptive and cyproterone acetate,
− OC+R subgroup—oral contraceptive and isotretinoin.

All patients in the study group received a contraceptive formulation of 0.03 µg ethinylestradiol and 3 mg drospirenone. The patients were then classified into one of the 3 subgroups described above: OC; OC+A; OC+R. In the OC+A group, an additional 50 mg of cyproterone acetate 1 × 1 for 10 days per treatment cycle was used for 3 months; as a reduction in acne severity was achieved, the cyproterone acetate dose was halved and used for another 3 months. In the OC+R group, isotretinoin was used at an initial dose of 10 mg for 3 months; as a partial reduction in the severity of acne was obtained, this treatment was continued with a dose of 20 mg for another 3 months.

Patients before treatment (T1) are defined as patients before the administration of acne therapy. Post-treatment (T2) patients are defined as patients after 6 months of follow-up.

#### 2.1.2. Inclusion and Exclusion Criteria

The general inclusion criteria for the study included: age 18–31 years, Caucasian women, normal history of puberty with no significant abnormalities on physical examination, not taking medication permanently, and patient consent to participate in the study.

Eligibility criteria for the study group included: the presence of acne in young women not previously treated for it. The control group included healthy young women without acne who attended the outpatient clinic for preventive examination and consented to biochemical tests.

Exclusion criteria from the study group were: disorders of growth and weight gain, endocrine diseases (e.g., thyroid disease, diabetes mellitus, polycystic ovary syndrome—PCOS, congenital adrenal hyperplasia—CAH, premature expiration of ovarian function—POF), which were diagnosed on the basis of history, gynecological examination and laboratory tests. Other exclusion criteria included participation in competitive sports, long-term use of stimulants, incomplete follow-up period, and use of another acne treatment regimen. Patients did not opt for cosmetic procedures or taking supplements to reduce sebum secretion (yeast pills, sulfur pills) and mattifying cosmetics were forbidden.

### 2.2. Instruments

All patients underwent a subjective and physical examination. Anthropometric measurements (height in cm, weight in kg) were taken and body mass index (BMI (kg/m^2^)) was calculated based on them.

The following parameters were determined from fasting venous blood taken in the morning: fasting glucose and insulin levels, total cholesterol (CHOL), LDL and HDL cholesterol, and triglycerides (TG). The tests were performed using electrochemiluminescence (ECLIA), enzyme-linked immunosorbent assay (ELISA), chemiluminescence-immunoassay (CLIA), and calorimetry.

The HOMA-IR insulin resistance index was calculated using HOMA2 Calculator software ©The University of Oxford (Oxford, UK) 2004–2021.

Visual assessment based on the Leeds scale was used to evaluate acne [19].

### 2.3. Statistical Methods

Statistical calculations were performed through the statistical computing environment (R v.4.1.1 (IDE RStudio v. 1.4.1717)). The significance level of statistical tests in this analysis was α = 0.05.

For variables on an interval scale, a description of the study set and drawing some basic conclusions and generalizations about the samples were conducted using grouped descriptive statistics. In addition, a normality test was carried out based on the Shapiro–Wilk test, including the W test statistic along with an indication of p significance.

Variables on a nominal, ordinal scale were analyzed in pairs in the form of contingency tables with an indication of frequency. The relationship of variables was examined using Fisher’s exact test; in addition, Cramer’s V measures of relationship strength were calculated.

For independent variables with a normal distribution with the number of independent groups above two, Welch’s one-way analysis of variance (ANOVA) was used to test the significance of differences. For two groups of independent samples with a normal distribution, the Welch’s *t*-test with Hedges’ g effect size calculation was used. For two groups of independent samples with a non-normal distribution, the Mann–Whitney U-test was used with a two-point correlation calculation based on ranks ${\hat{r}}_{biserial}^{rank}$.

For two dependent variables with a normal distribution, the Student’s t test with Hedges’ g effect size calculation was used to test the significance of differences. For two dependent variables with a normal distribution, the non-parametric Wilcoxon paired rank-sum test was used. The value of the association between the variables was calculated using rank-based two-point correlation ${\hat{r}}_{biserial}^{rank}$.

In the case of examining the correlation between variables on the ordinal scale and the quotient scale, the Kendall rank correlation coefficient τb was calculated in the form of an appropriate measure of the relationship.

In the case of calculating the correlation between a variable on an ordinal scale and a variable on a nominal (dichotomous) scale in the form of a correlation coefficient, a two-point correlation based on ranks was estimated, ${\hat{r}}_{biserial}^{rank}$. When the ordinal variable has only two levels, Yule’s phi linkage measure (φc) was used.

### 2.4. Ethics

The study was conducted in accordance with the Declaration of Helsinki and approved by the Ethics Committee of the Pomeranian Medical University in Szczecin, Poland (protocol code KB-012/78/18 on 18 June 2018).

## 3. Results

### 3.1. Descriptive Statistics of Variables on an Interval Scale

The study participants (study group and control group) were not statistically significantly different in terms of the study variables shown in Table 1.

Significant differences were found in insulin (*p* = 0.001), cholesterol (*p* = 0.034), HDL (*p* = 0.030), and HOMA IR (*p* = 0.001) concentrations between the pre-treatment study group and the control group. The remaining concentration differences were not significant. Detailed characteristics of concentrations in the pre-treatment study group and the control group are shown in Table 2.

### 3.2. Relationships between Metabolic Factors and Diet and Acne Severity before Treatment

There was a significant correlation between LDL concentration and the Leeds scale (*p* = 0.018) and between sweet consumption and the Leeds scale (*p* = 0.034). The remaining correlations were not significant. Detailed correlations of the study variables with the Leeds scale are shown in Table 3.

### 3.3. Leeds Acne Severity Score before and after Treatment

It was found that the severity of acne in the study group without division by treatment type was significantly lower after treatment when compared to before treatment (*p* = 0.001). The data are shown in Table 4.

The severity of acne on the Leeds scale before and after treatment was also compared, taking into account the type of treatment used:

− Treatment regimen: oral contraception (OC)

A Wilcoxon paired rank-sum test of the dependent variables showed a significant reduction in the severity of acne on the Leeds scale from Mdn = 3.00, IQR = 0.00 before treatment to Mdn = 1.00, IQR = 0.00 after treatment with the contraceptive, V_Wilcoxon_ = 561.00, *p* < 0.001. The measure of association was estimated as “large”, ${\hat{r}}_{biserial}^{rank}$ = 1.0.

− Treatment regimen: oral contraception (OC) + cyproterone acetate (A)

The Wilcoxon paired rank-sum test for the dependent variables showed a significant reduction in the free androgen index (FAI) from Mdn = 1.45, IQR = 1.01 before treatment to Mdn = 0.23, IQR = 0.11 after treatment with the contraceptive along with cyproterone acetate, V_Wilcoxon_ = 496.00, *p* < 0.001. The measure of association was estimated as “large”, ${\hat{r}}_{biserial}^{rank}\;$= 1.0.

− Treatment regimen: contraception (OC)+ isotretinoin (R)

The Wilcoxon paired rank-sum test for the dependent variables showed a significant reduction in the free androgen index (FAI) from Mdn = 3.00, IQR = 1.00 before treatment to Mdn = 1.00, IQR = 2.00 after treatment with the contraceptive formulation along with the isotretinoin formulation, V_Wilcoxon_ = 528.00, *p* < 0.001. The measure of association was estimated as “large”, ${\hat{r}}_{biserial}^{rank}$= 1.0.

**Figure 1:**
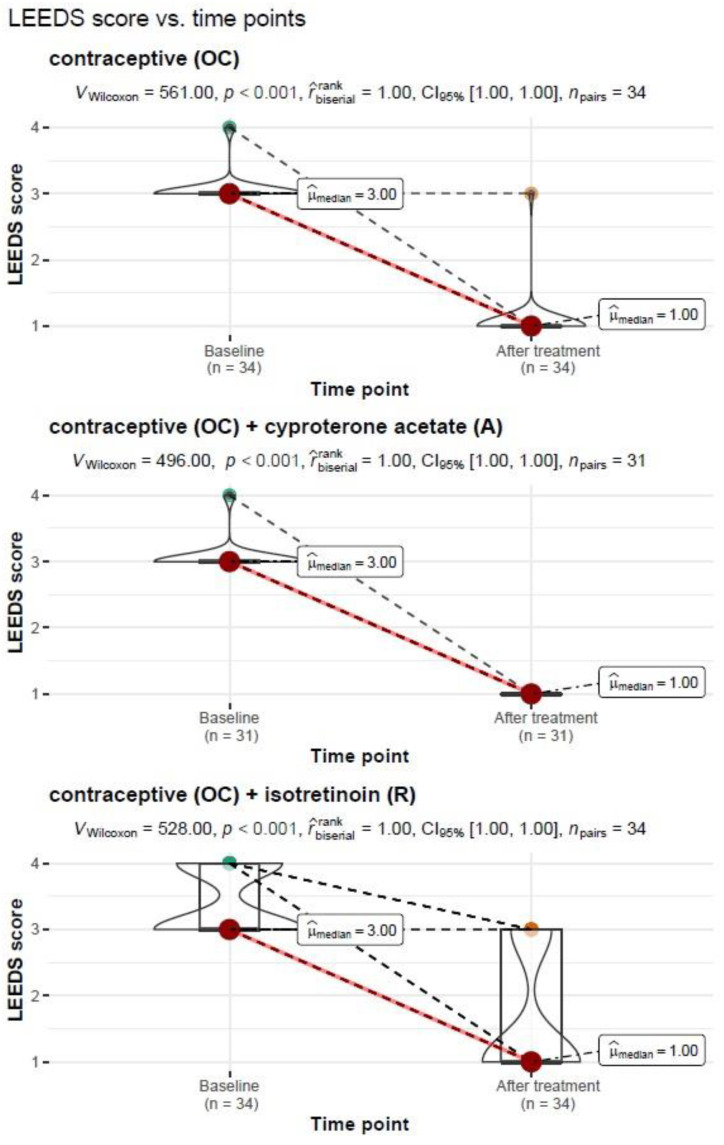
Comparison of median measures of acne severity on the Leeds scale before and after treatment by type of treatment performed.

A graphical representation of the comparison of median measures of acne severity on the Leeds scale along with expanded statistical test reporting by treatment type is shown in Figure 1.

### 3.4. Relationships between Acne Severity on the Leeds Scale and Consumption of Dairy and Sweets in the Study Group

The relationship between nominal factors of dairy and sweets intake, and treatment outcome was examined using contingency tables. Treatment outcome was estimated as the difference in Leeds scores after treatment and before treatment. In addition, the relationship between the variables was examined separately based on the type of treatment.

#### 3.4.1. Leeds vs. Dairy Consumption Factor

##### Subgroup Treated with Oral Contraception

The distribution of the Leeds acne treatment outcome variable after and before treatment with the contraceptive vs. dairy intake factor is shown in Table 5.

Fisher’s test of independence conducted showed no significant relationship between the difference in acne severity levels on the Leeds scale before and after treatment with contraception and the factor of dairy consumption (*df* = 3, *p* = 0.361). The association between the variables was estimated as “moderate,” *V* = 0.33.

##### Subgroup Treated with Oral Contraception and Cyproterone Acetate

The distribution of the variable acne treatment score on the Leeds scale before and after treatment with a contraceptive along with cyproterone acetate vs. dairy intake factor is shown in Table 6.

Fisher’s test of independence showed no significant relationship between the difference in acne severity levels on the Leeds scale before and after treatment with a contraceptive formulation along with cyproterone acetate and the factor of dairy consumption (*df* = 3, *p* = 0.462). The association between the variables was estimated as “moderate,” *V* = 0.27.

##### Subgroup Treated with Oral Contraception and Isotretinoin

The distribution of the variable Leeds acne treatment score before and after treatment with the contraceptive along with isotretinoin vs. dairy intake factor is shown in Table 7.

Fisher’s test of independence showed no significant relationship between the difference in acne severity levels on the Leeds scale before and after treatment with a contraceptive formulation along with an isotretinoin formulation and the factor of dairy consumption (*df* = 3, *p* = 0.362). The association between the variables was estimated as “faint,” *V* = 0.33.

#### 3.4.2. Leeds vs. Sweets Consumption Factor

##### Subgroup Treated with Oral Contraception

The distribution of the Leeds acne treatment outcome variable before and after treatment with the contraceptive vs. sweets intake factor is shown in Table 8.

Fisher’s test of independence showed no relationship between the difference in acne severity levels on the Leeds scale before and after treatment with the contraceptive and the factor of sweets consumption (*df* = 3, *p* = 1.000). The association between the variables was estimated as “faint,” *V* = 0.05.

##### Subgroup Treated with Oral Contraception and Cyproterone Acetate

The distribution of the variable Leeds acne treatment score before and after treatment with a contraceptive along with cyproterone acetate vs. a factor of sweets consumption is shown in Table 9.

Fisher’s test of independence showed no significant relationship between the difference in acne severity on the Leeds scale before and after treatment with the contraceptive along with cyproterone acetate and the factor of sweets consumption (*df* = 3, *p* = 0.191). The association between the variables was estimated as “moderate,” *V* = 0.34.

##### Subgroup Treated with Oral Contraception and Isotretinoin

The distribution of the variable Leeds acne treatment score before and after treatment with a contraceptive formulation along with an isotretinoin formulation vs. the sweets intake factor is shown in Table 10.

Fisher’s test of independence showed no significant relationship between the difference in acne severity levels on the Leeds scale before and after treatment with a contraceptive along with isotretinoin and the factor of sweet consumption (*df* = 3, *p* = 0.510). The association between the variables was estimated as “moderate,” *V* = 0.34.

## 4. Discussion

The results obtained in this article regarding metabolic factors showed statistical significance in terms of insulin concentrations and HOMA index, which were higher in the study group with acne compared to the control group. Recently, there have been reports on the possible influence of excess insulin on androgen metabolism in the adrenal glands and gonads primarily during adolescence; this can exacerbate acne lesions. Confirmation of the presented results is provided by Emiroglu et al., who compared glucose and insulin concentrations in 243 female patients with severe acne to 156 healthy controls. They found that while fasting glucose levels were not significantly different between the groups, fasting insulin levels were significantly higher in the diseased group; in addition, the HOMA index was higher in those with acne [20]. In another study, Kartal et al. found elevated levels of insulin, glucose, and HOMA index in 46 female subjects with acne when compared to a group of healthy women. Elevated serum glucose and insulin levels correlated with sebum overproduction and the severity of acne lesions [21]. Similarly, Cappel et al. showed that elevated levels of insulin in adult women and men is more likely to accompany acne lesions [22]. A study by Vora et al. showed a positive correlation between the amount of sebum secreted on the face and insulin levels in people with acne [23]. Another study describing this issue showed that the frequency of IGF-1 genotype (CA) 19 was significantly different between control and acne patients (*p* = 0.0002). This study found a significant association between IGF-I (CA) genotypes and acne severity (*p* = 0.015). There was no significant difference between male and female patients (*p* > 0.05). The results suggest that the IGF-I (CA) 19 polymorphism may contribute to a predisposition to acne in Turkish patients [24]. Conflicting reports were cited by Balta et al., who evaluated a group of 35 patients with post-adolescent acne. The authors compared the results of glucose metabolism to 35 healthy control subjects; they showed no differences in glucose, insulin, and HOMA index values between the groups [25]. Facts worth mentioning concern the analysis of the causes of androgen excess in women with obesity. It seems that the mechanism described in polycystic ovary syndrome associated with excess visceral fat and reduced insulin sensitivity leading to insulin resistance and high blood insulin concentrations comes to the fore. Insulin resistance in peripheral tissues and hyperinsulinemia leads not only to the activation of glucose transport pathways in adipose and muscle tissue but also, selectively, to the activation of testosterone production in the ovaries and an increase in its concentration in the blood. Some of the androgens also come from the increased peripheral conversion of estradiol to testosterone in adipose tissue in obesity.

Hyperinsulinemia also affects the inhibition of hepatic SHBG synthesis, thereby increasing free testosterone concentrations. Evidence for the key role of insulin in androgen production in obesity and hyperinsulinemia is the significant reduction in testosterone and androstendione concentrations, following weight reduction, decreased insulin levels, and improved insulin sensitivity in patients treated with diet and pharmacological therapy [26,27,28]. These observations provide a clinical indicator for promoting normal body weight and lifestyle changes in female patients with acne. The so-called “Western” diet, characterized by increased dairy consumption and a high glycemic index (GI), has been shown to affect the levels of hormones involved in the pathogenesis of acne [29]. In contrast, this study shows that consumption of sweets correlates with acne severity. In ongoing studies, high-GI diets have been associated with impaired carbohydrate tolerance and higher postprandial insulin concentrations and elevated levels of insulin-like growth factor 1 (IGF-1), while low-GI diets have been shown to reduce fasting IGF-1 concentrations [29,30]. Based on these studies, many research groups have wondered whether consuming dairy or high-GI foods has an effect on modulating the development and severity of acne. Moreover, patients often ask whether dietary changes can improve their skin condition. As a result, interest in the topic has increased in recent years and the literature is constantly supplemented with new studies [30,31].

Akpinar Kara Y et al. conducted a study involving 53 patients with acne vulgaris and 53 age-, gender- and ethnicity-matched controls. They showed that cheese and carbohydrate intake was higher in the acne group compared to the control group (*p* < 0.05). In addition, they observed that cheese consumption increased acne formation and carbohydrate consumption increased severity, while fat consumption was not significant [32]. In a cross-sectional analysis of a large cohort of 24,452 participants in the French NutriNet-Santé study, showed that the consumption of fatty and sugary foods, sweet drinks, and milk was associated with acne in adults. These results were adjusted for potential confounding variables (age, gender, physical activity, smoking, education level, daily energy intake, number of completed dietary records, and depressive symptoms). The results of this study support the hypothesis that the so-called Western diet (rich in animal products and fatty and sugary foods) is associated with the incidence of acne in adulthood [33]. A high glycemic load diet causes an increase in circulating IGF-1 and insulin levels. IGF-1 levels also stimulate the production of androgens, which are associated with sebum production and thus the development of acne [34]. Consumption of milk also increases hepatic IGF-1 production and circulating insulin levels; moreover, neither IGF-1 nor insulin are fully inactivated by pasteurization, homogenization, and digestion [35]. Hence, consuming milk has similar consequences to a high glycemic load meal [36]. Other diverse randomized studies that do or do not confirm the effect of diet on acne severity are less reliable because they did not examine multiple food exposures during the same period and are limited by a small population sample. In addition, observational studies often focus on adolescent acne and rely on frequency questionnaires on previous food exposures, leading to both a lack of precision in food records and recall bias without considering various potential confounders like depression or smoking [37,38,39]. On this basis, this article assumes that diet can influence acne in selected populations. Increased carbohydrate intake and a high-GI diet promote and exacerbate acne symptoms, while increased dairy intake promoted acne formation only in Western populations [33].

Another analysis in the present article looked at the severity of acne after treatment in the study group and subgroups (OC; OC+A; OC+R). In all study groups with and without treatment, the degree of acne severity after treatment was significantly lower, i.e., there was clinical improvement (*p* < 0.001). Thus, it can be assumed that each treatment option had a beneficial effect on the skin in the study group of female patients. It can be assumed that this was due to the contraceptive preparation. None of the treatment options used in the short 6 months of use was the most beneficial. According to the recommendations of dermatologists, treatment is applied depending on the severity of the lesions and the effectiveness of the preparations used. Therefore, a thorough evaluation of acne before treating patients is essential [40,41].

The literature shows that elevated androgen levels are present in about 50% of patients; in addition, about 50% to 60% of adult women with acne and normal androgen levels show elevated levels of androgen metabolites, suggesting some form of androgen excess. Therefore, most acne treatment recommendations involve the use of anti-androgen drugs [42,43]. According to the recommendations of the American Academy of Dermatology, oral estroprogestogen or anti-androgen therapy is used as second-line treatment for women with moderate to severe adult acne who do not respond to local therapy and antibiotic therapy, regardless of androgen levels (there are no recommendations for measuring androgens in women with adult acne [15]). European dermatologists use hormonal treatment in patients with mild acne if hyperandrogenism is found, and for moderate acne without signs of androgen excess. In all patients, treatment with estroprogestogens or antiandrogens is used along with local acne treatment [44]. From the point of view of the AE-PCOS Task Force, it is important to treat hyperandrogenism regardless of acne severity if there is clinical or biological evidence for it [33]. In addition, the team, after careful evaluation of all available data, made recommendations that indicated that the diagnosis of adult female acne is primarily clinical, and that serum androgen levels (total testosterone, free testosterone, and DHEAS) should be determined in all women with adult acne after excluding other diseases. All second- and third generation estroprogestagens can be used, regardless of the dose of estrogen and progestogen component. Importantly, the authors noted that estroprogestagens can be used in non-hyperandrogenemic patients with adult acne as second-line therapy when local treatment has failed [33].

Understanding the skin pathology associated with androgenization is important for gynecologists, dermatologists, endocrinologists, family physicians, pediatricians, and cosmetologists. Early diagnosis and treatment will help prevent complications of acne vulgaris.

Our study also had limitations. The main limitation was the six month observation period which, from the perspective of the preparations used, did not allow us to assess the long-term effect of acne treatment in the subgroups. However, we are continuing the study to identify these long-term effects. Another limitation may be related to deficiencies in the obtained results, which is the reason for the different number of variables in some correlations. Some of the patients did not complete all the recommended test results. However, the missing values were for side parameters that did not form the core of this work, so, despite this, we decided to exclude patients from the analysis. The patients’ use of several local potential determinants affecting acne severity and the Leeds scale scores cannot be excluded. Nevertheless, due to the appropriate choice of statistical methodology and the large sample size, our findings are reliable, and the assumptions are supported by their substantial consistency with published evidence. There were also concerns about the existing measures of acne severity and the inadequacy of existing acne severity scales. To address this concern, we conducted a critical review of the originally published acne scales to formally assess their quality based on a set of predetermined criteria. The lack of an internationally accepted measure of acne severity hinders high-quality clinical trials and the adoption of best practices with potential consequences for the acne sufferer. We conclude, as others have, that a robust scoring system for assessing acne severity is required. Future development of acne scales or further study of currently available scales is needed to correct these problems and move closer to developing a valid and reliable “gold standard” tool.

## 5. Conclusions

LDL levels and consumption of sweets correlate with the severity of acne. The mainstay of acne treatment is contraceptive treatment (ethinylestradiol and drospirenone). The effectiveness of the three contraceptive-based treatments was confirmed by observing the severity of acne. There were no significant relationships between the difference in acne severity before and after treatment with the three treatment regimens and factors of dairy or sweets consumption.

## Figures and Tables

**Table 1:** Characteristics of study participants including age, weight, height, and BMI.

Variable	Group	N	M	SD	Mdn	IQR	Min	Max	*p*
Age	Study	99	25.93	5.95	25.00	9.00	18.00	31.00	0.6122
	Control	69	23.84	1.15	24.00	1.00	21.00	28.00	
Weight	Study	99	67.29	14.02	63.00	12.50	54.00	89.00	0.6688
	Control	69	60.46	7.06	60.00	9.00	58.00	85.00	
Height	Study	99	163.07	13.13	164.00	8.50	158.00	173.00	0.1949
	Control	69	164.58	5.45	164.00	8.00	155.00	176.00	
BMI	Study	99	25.00	5.00	24.00	5.05	18.69	30.72	0.5312
	Control	69	22.28	1.94	22.31	2.34	18.42	28.40	

N—sample size; M—mean; SD—standard deviation; Mdn—median; IQR—interquartile range; Min—minimum; Max—maximum; *p*—*p*-value.

**Table 2:** Characteristics of selected metabolic parameters in women in the study group (before treatment) compared to the control group.

Variable	Group	N	M	SD	Mdn	IQR	Min	Max	*p*
glucose	Study	99	88.26	7.54	88.90	10.40	70.70	107.00	0.119
	Control	69	86.78	7.34	87.00	10.30	70.70	107.20	
insulin	Study	99	10.23	4.95	9.20	5.95	1.50	32.30	0.001
	Control	69	11.19	10.82	8.81	6.38	1.71	90.10	
HOMA IR	Study	99	1.40	0.67	1.29	0.84	0.37	4.08	0.001
	Control	69	1.28	0.62	1.14	0.81	0.38	3.53	
cholesterol	Study	99	174.33	27.25	173.50	36.05	73.30	237.20	0.034
	Control	69	185.73	26.07	181.90	33.60	130.00	283.40	
HDL	Study	99	71.92	14.94	71.30	20.00	33.00	116.30	0.030
	Control	69	74.26	16.75	74.70	20.20	4.50	111.80	
LDL	Study	99	99.57	21.79	98.40	31.85	53.20	167.20	0.201
	Control	69	101.38	22.52	98.60	32.80	58.70	182.70	
triglycerides	Study	99	80.04	34.04	73.50	42.80	33.80	271.50	0.6321
	Control	69	78.49	29.61	72.70	39.20	33.80	188.50	

N—sample size; M—mean; SD—standard deviation; Mdn—median; IQR—interquartile range; Min—minimum; Max—maximum; *p*—*p*-value.

**Table 3:** Correlation coefficients between metabolic factors and diet and acne severity on the Leeds scale before treatment.

Variables	Leeds Scale	
	Correlations (τ_b,_ φ_c_)	*p*
BMI	0.04	0.673
glucose	0.03	0.693
insulin	0.09	0.276
HOMA IR	0.07	0.414
cholesterol	0.09	0.263
HDL	0.07	0.372
LDL	0.18	0.018
triglycerides	0.01	0.895
dairy consumption	0.03	0.786
sweet consumption	0.09	0.034

**Table 4:** Acne severity scores of the study group before treatment (T1) and after treatment (T2) based on the Leeds scale.

Variable	Group	n	M	SD	Mdn	IQR	Min	Max	*p*
Leeds	Study _T1_	99	3.18	0.39	3.00	0.00	3.00	4.00	0.001
Leeds	Study _T2_	99	1.22	0.44	1.00	0.00	1.00	3.00	

**Table 5:** Frequencies (%) of the distribution of the difference in acne severity on the Leeds scale after and before treatment vs. dairy intake factor in the treatment group with contraception (N = 34).

Δ Leeds ^1^	Dairy Consumption Factor		
	No Consumption	Consumption	Total
−3	3 (50%)	3 (50%)	6
−2	11 (61.1%)	7 (38.9%)	18
−1	7 (87.5%)	1 (12.5%)	8
0	2 (100%)	0	2
Total	23 (67.6%)	11 (32.4%)	34

^1^ Leeds after treatment—Leeds before treatment [points].

**Table 6:** Frequencies (%) of the distribution of the difference in acne severity on the Leeds scale before and after treatment vs. dairy intake factor in the group treated with the contraceptive formulation along with cyproterone acetate formulation (N = 31).

Δ Leeds ^1^	Dairy Consumption Factor		
	No Consumption	Consumption	Total
−3	1 (50%)	1 (50%)	2
−2	22 (75.9%)	7 (24.1%)	29
−1	0	0	0
0	0	0	0
Total	23 (74.2%)	8 (25.8%)	31

^1^ Leeds after treatment—Leeds before treatment [points].

**Table 7:** Frequencies (%) of the distribution of the difference in acne severity on the Leeds scale before and after treatment vs. dairy intake factor in the group treated with the contraceptive along with isotretinoin (N = 34).

Δ Leeds ^1^	Dairy Consumption Factor		
	No Consumption	Consumption	Total
−3	3 (50%)	3 (50%)	6
−2	11 (61.1%)	7 (38.9%)	18
−1	7 (87.5%)	1 (12.5%)	8
0	2 (100%)	0	2
Total	23 (67.6%)	11 (32.4%)	34

^1^ Leeds after treatment—Leeds before treatment [points].

**Table 8:** Frequencies (%) of the distribution of the difference in acne severity on the Leeds scale before and after treatment vs. sweets intake factor in the treatment group with contraception (N = 34).

Δ Leeds ^1^	Sweets Consumption Factor		
	No Consupmtion	Consumption	Total
−3	2 (100%)	0	2
−2	26 (83.9%)	5 (16.1%)	31
−1	0	0	0
0	1	0	1
Total	29 (85.3%)	5 (14.7%)	34

^1^ Leeds after treatment—Leeds before treatment [points].

**Table 9:** Frequencies (%) of the distribution of the difference in acne severity on the Leeds scale before and after treatment vs. the sweets intake factor in the group treated with the contraceptive along with cyproterone acetate (N = 31).

Δ Leeds ^1^	Sweets Consumption Factor		
	No Consumption	Consumption	Total
−3	1 (50%)	1 (50%)	2
−2	27 (93.1%)	2 (6.9%)	29
−1	0	0	0
0	0	0	0
Total	28 (90.3%)	3 (9.7%)	31

^1^ Leeds after treatment—Leeds before treatment [points].

**Table 10:** Frequencies (%) of the distribution of the difference in the severity of acne on the Leeds scale after and before treatment vs. the sweets intake factor in the group treated with the contraceptive along with isotretinoin (N = 34).

Δ Leeds ^1^	Sweets Consumption Factor		
	No Consumption	Consumption	Total
−3	6 (100%)	0	6
−2	14 (77.8%)	4 (22.2%)	18
−1	8 (100%)	0	8
0	2	0	2
Total	30 (88.2%)	4 (11.8%)	34

^1^ Leeds after treatment—Leeds before treatment [points].

## Data Availability

The data presented in this study are available on request from the author E.S.-P. The data are not publicly available due to ethical restrictions.
